# Silicates Eroded under Simulated Martian Conditions Effectively Kill Bacteria—A Challenge for Life on Mars

**DOI:** 10.3389/fmicb.2017.01709

**Published:** 2017-09-12

**Authors:** Ebbe N. Bak, Michael G. Larsen, Ralf Moeller, Silas B. Nissen, Lasse R. Jensen, Per Nørnberg, Svend J. K. Jensen, Kai Finster

**Affiliations:** ^1^Department of Bioscience, Aarhus University Aarhus, Denmark; ^2^Space Microbiology Research Group, Institute of Aerospace Medicine, German Aerospace Center (DLR) Cologne, Germany; ^3^Department of Chemistry, Aarhus University Aarhus, Denmark; ^4^Stellar Astrophysics Center, Department of Physics and Astronomy, Aarhus University Aarhus, Denmark

**Keywords:** habitability, erosion, reactive oxygen species, forward contamination, stress factors, saltation, toxicity, microorganisms

## Abstract

The habitability of Mars is determined by the physical and chemical environment. The effect of low water availability, temperature, low atmospheric pressure and strong UV radiation has been extensively studied in relation to the survival of microorganisms. In addition to these stress factors, it was recently found that silicates exposed to simulated saltation in a Mars-like atmosphere can lead to a production of reactive oxygen species. Here, we have investigated the stress effect induced by quartz and basalt abraded in Mars-like atmospheres by examining the survivability of the three microbial model organisms *Pseudomonas putida, Bacillus subtilis*, and *Deinococcus radiodurans* upon exposure to the abraded silicates. We found that abraded basalt that had not been in contact with oxygen after abrasion killed more than 99% of the vegetative cells while endospores were largely unaffected. Exposure of the basalt samples to oxygen after abrasion led to a significant reduction in the stress effect. Abraded quartz was generally less toxic than abraded basalt. We suggest that the stress effect of abraded silicates may be caused by a production of reactive oxygen species and enhanced by transition metal ions in the basalt leading to hydroxyl radicals through Fenton-like reactions. The low survivability of the usually highly resistant *D. radiodurans* indicates that the effect of abraded silicates, as is ubiquitous on the Martian surface, would limit the habitability of Mars as well as the risk of forward contamination. Furthermore, the reactivity of abraded silicates could have implications for future manned missions, although the lower effect of abraded silicates exposed to oxygen suggests that the effects would be reduced in human habitats.

## Introduction

An in-depth understanding of the interaction between the Martian surface environment and living cells is essential for assessment of the habitability of Mars, the risk of forward contamination and the hazards associated with manned missions. The Martian surface temperatures vary from −150° to 20°C, and the surface pressure is usually 4–10 mbar (Millour et al., 2015). Consequently, the Martian surface is extremely arid and even if liquid water is present, the water activity is likely too low to support life (Martin-Torres et al., 2015; Ojha et al., 2015). While such an arid, cold, low-pressure environment often is not detrimental for desiccation tolerant, dormant cells and spores, the radiation on the Martian surface can be highly damaging (Hansen et al., 2009; Johnson et al., 2011). The solar irradiation flux on Mars is about 40% of the irradiation on Earth, but, the thin atmosphere and the sparse ozone layer (Lefevre et al., 2008) result in a substantially higher UVB and UVC radiation than on Earth (Cockell et al., 2000). Schuerger et al. (2003) demonstrated that 15 min of exposure to Mars-like UV radiation sterilized monolayers of *Bacillus subtilis* spores. The effect of UV radiation is, however, reduced dramatically when cells and spores are shielded by a thin layer of dust or a few layers of dead cells (Mancinelli and Klovstad, 2000; Diaz and Schulze-Makuch, 2006; de La Vega et al., 2007; Paulino-Lima et al., 2010). The flux of ionizing radiation from the sun and galactic cosmic rays are three orders of magnitude higher on the Martian surface as compared to the surface of Earth due to the thin Martian atmosphere and the absence of a global magnetic field (Hassler et al., 2014). Nevertheless, ionizing radiation from the sun only poses a minor challenge for *B. subtilis* and *D. radiodurans*, and a cover that would protect against UV radiation would also shield from the charged particles in the solar wind (Tuleta et al., 2005; Paulino-Lima et al., 2011). Protection against galactic cosmic rays, mainly high-energy particles, requires much thicker shielding. However, the flux to the Martian surface would only cause a half-life of *B. subtilis* spores within the order of thousands of years (Moeller et al., 2010; Hassler et al., 2014). In summary, the Martian surface does not generally allow life to proliferate, but may not pose an immediate threat to dormant cells.

The Martian soil is dominated by silicate minerals (Bish et al., 2013) primarily produced by physical abrasion of olivine basalts (Morris et al., 2004; Gunnlaugsson et al., 2009). The soil and dust particles are produced by meteor impacts and by wind-driven saltation through repetitive low-energy collisions (Kok et al., 2012). A recent study showed that silicates exposed to simulated wind-driven saltation in a Mars-like atmosphere could lead to a production of reactive oxygen species (ROS) including hydrogen peroxide (H_2_O_2_) and hydroxyl radicals (·OH) (Bak et al., 2017). A production of ROS from abraded silicates may explain the oxidative capabilities of the Martian soil as observed by the Viking Biological Experiments (Klein, 1978) and may cause oxidative stress for living cells. Based on these observations, we hypothesized that wind abraded silicates on Mars may pose an additional stress factor for living cells.

To test our hypothesis, we conducted a series of experiments in which cell suspensions of *Pseudomonas putida, B. subtilis* and *D. radiodurans* were exposed to quartz and basalt samples abraded by simulated wind-driven saltation in Mars-like atmospheres. The exposure experiments were conducted without exposing the abraded material to oxygen to simulate the *in situ* conditions for indigenous organisms and a scenario of forward contamination. Furthermore, we investigated the effect of secondary exposure to air to simulate the environment inside a human habitat. *Pseudomonas putida* is a common soil bacterium, which can withstand some level of oxidative stress (Kim and Park, 2014). The endospores of *B. subtilis* and cells of *D. radiodurans* have been reported to be highly resistant against a range of stress factors including desiccation, chemical oxidative stress (Melly et al., 2002; Slade and Radman, 2011) and ionizing radiation (Anderson et al., 1956; Moeller et al., 2008). Furthermore, they have been used to study the effects of exposure to a Mars-like environment including the impact of UV and ionizing radiation (Diaz and Schulze-Makuch, 2006; de La Vega et al., 2007; Moeller et al., 2010; Kerney and Schuerger, 2011). The resistance of *B. subtilis* to abraded silicates was further investigated by testing a range of mutant strains lacking specific spore components, which previously have been found to be related to resistance against oxidizing agents (Setlow, 2014). *Bacillus sp*. spores are of particular interest and concern, as they are common contaminants in cleanrooms used for assembly of spacecraft (Puleo et al., 1977; La Duc et al., 2009; Smith et al., 2017) and thus likely agents of forward contamination.

## Materials and methods

### Simulated saltation of silicates

**Figure 1:**
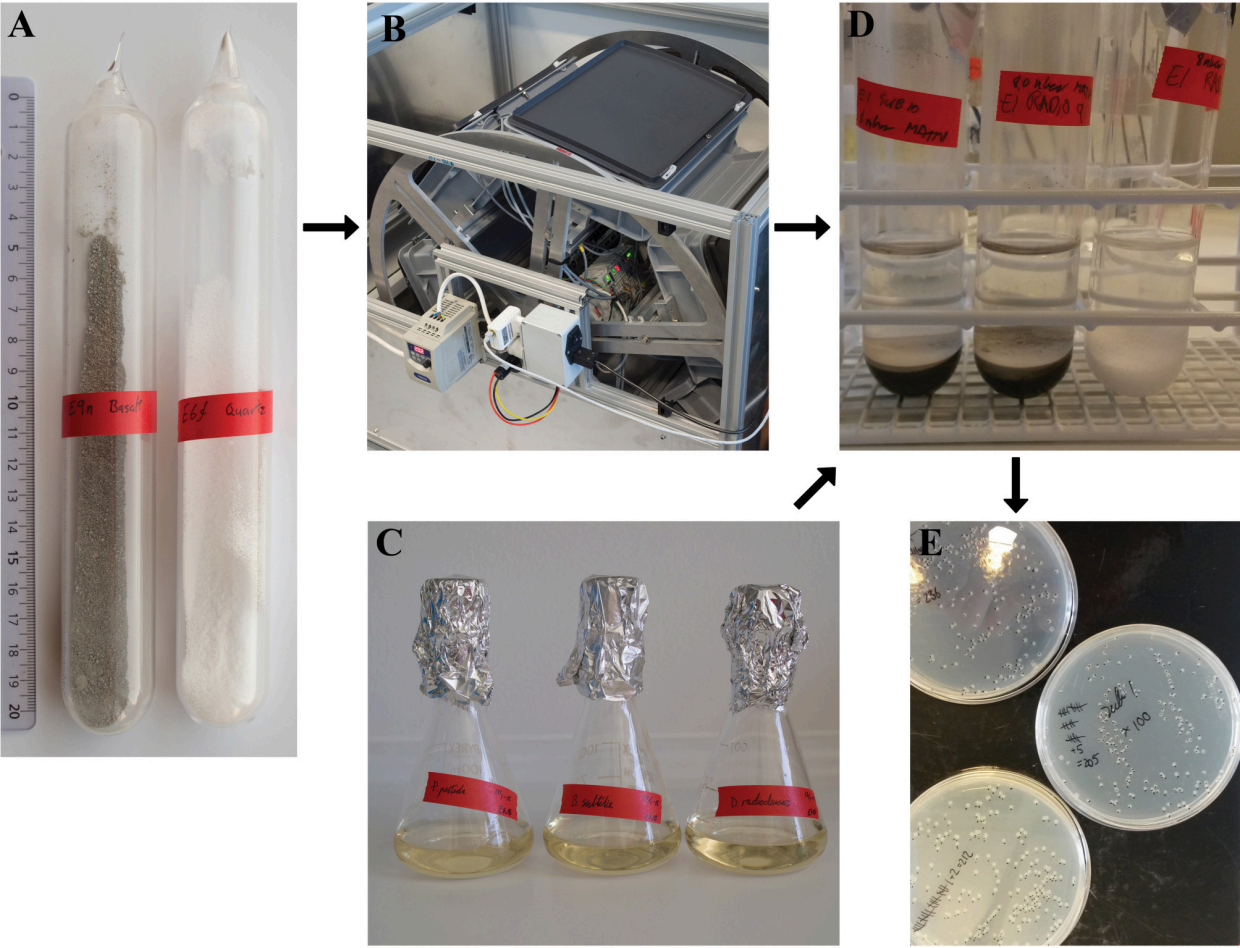
**(A)** Sealed quartz ampoules with basalt (left) and quartz sand (right). **(B)** Turning wheel running at 30 rpm. The ampoules were fixed inside the gray boxes. **(C)** Media inoculated with cell cultures. **(D)** Abraded silicate samples mixed with washed cell cultures. **(E)** Agar plates with colonies of the tested bacteria.

Quartz sand samples were prepared by sieving commercially available quartz (Merck, Cat. No. 1.07536) to obtain the 125–1,000 μm fraction. The quartz sand was washed and dried to remove small particles and finally divided into 10 g aliquots using a Fritsch Rotary cone samples divider. The same procedure was used to produce 10 g basalt aliquots based on olivine basalt that was collected in Gufunes on Iceland (64°08′22.18″N, 21°47′21.27″W) and crushed. The mineral composition of the same batch of crushed basalt was reported in Bak et al. (2017). The samples were transferred to about 20 cm long and 3 cm wide quartz ampoules with rounded ends (Figure 1A). One end of the ampoules was extended by a narrow quartz inlet tube that allowed connection to a vacuum system. The ampoules were evacuated to <0.12 mbar and filled with 8 ± 0.2 mbar of a Mars-like atmosphere composed of 95% CO_2_ (>99.9% purity, AGA, Denmark) and 5% of a custom made gas mixture (>99% purity, Air Liquid, Denmark) to give a final composition of 95% CO_2_, 3% N_2_, 1.75% Ar, 0.15% O_2_ and 0.1% CO, which is similar to the composition of the Martian atmosphere (Mahaffy et al., 2013). The pressure was monitored with a Pfeiffer TPR 265 Pirani gauge for *p* < 1 mbar and a Pfeiffer APR 250 Pirani gauge for *p* > 1 mbar. The ampoules were sealed by melting off the narrow inlet tubes. Wind-driven saltation was simulated by mounting the ampoules in a turning wheel running at 30 rpm (Figure 1B). This set-up caused the silicate samples to fall from one end of the ampoules to the other once per second, which mimics the repeated low energy impacts that are characteristic for saltation (Merrison, 2012). The samples were tumbled for 63 days, which led to a considerable abrasion of the material resulting in an increase of the specific surface area of 3.44 and 0.76 m^2^ g^−1^ for the quartz and the basalt samples, respectively (Bak et al., 2017). Some of the abraded samples were suspended in 10 ml of water followed by overnight drying at 60°C, which has been shown to neutralize the potential for production of ROS (Bak et al., 2017). These samples were used as negative controls and are hereafter referred to as inactivated samples.

### Preparation of cell suspensions

The bacterial strains used in this study are listed in Table 1. *Pseudomonas putida* mt-2 KT2440, *Bacillus subtilis* 168 and *Deinococcus radiodurans* R1 were obtained from the German Collection of Microorganisms and Cell Cultures GmbH (DSMZ, Braunschweig, Germany) as DSM6125, DSM402 and DSM20539, respectively. Stock cultures of all strains were stored as aliquots in 15% glycerol at -80°C. The following growth media and buffer solutions were used: Lysogeny Broth (LB) medium [10 g NaCl, 10 g tryptone (Fluka) and 5 g yeast extract (Merck) per liter, pH adjusted to 7]; Nutrient Broth (NB) medium (Scharlau) and phosphate buffered saline (PBS) (8 g NaCl, 0.2 g KCl, 1.44 g Na_2_HPO_4_ and 0.24 g KH_2_PO_4_ per liter, pH adjusted to 7.3). For preparation of agar plates the liquids were supplemented with 1.5% w/w of bacteriological agar. All chemicals were of analytical grade and acquired from commercial suppliers. MilliQ water was used for all solutions. *P. putida* cultures were grown in 100 ml Erlenmeyer flasks containing 30 ml of medium composed of 20% LB-medium and 80% PBS. *Bacillus subtilis* and *D. radiodurans* were grown under the same condition, but in a medium composed of 20% NB-medium and 80% PBS (Figure 1C). Pre-cultures were prepared from frozen stocks and incubated at room temperature (21–23°C) on a shaker at 120 rpm. The optical density (OD) was measured after ~24 h at 600 nm and the equivalent of 1 ml of OD_600_ = 0.1 was transferred to fresh medium and incubated under identical conditions. The survival experiments were carried out with cultures that were harvested in the late exponential growth phase (after 10–12 h for *P. putida* and *B. subtilis* and 17–19 h for *D. radiodurans*) and in the stationary phase (after 118–120 h for all species), see Figure S1. The purity of the cultures was checked by microscopy.

**Table 1:** Bacterial strains used in this study.

**Strain**	**Genotype and/or phenotype^a^**	**Source (References)**
***Pseudomonas putida* STRAIN**		
DSM6125	Wild type (mt-2 KT2440)	DSMZ^b^
***Deinococcus radiodurans* STRAIN**		
DSM20539 (type strain)	Wild type (R1)	DSMZ
***Bacillus subtilis* STRAINS**		
DSM402	Wild type (168)	DSMZ
PS832	Wild type parent of PS356	P. Setlow (Slieman and Nicholson, 2001)
PY79 (PE594)	Wild type parent of PE277, PE618, PE620, and PE1720, prototroph	P. Eichenberger (Raguse et al., 2016)
PE277	*safA::tet*, Tet^R^	P. Eichenberger (Raguse et al., 2016)
PE618	*cotE::cat*, Cm^R^	P. Eichenberger (Raguse et al., 2016)
PE620	*cotX cotYZ::neo*, Neo^R^	P. Eichenberger (Raguse et al., 2016)
PE1720	*safA::tet*, Tet^R^ *cotE::cat*, Cm^R^	P. Eichenberger (Raguse et al., 2016)
PS356	*sspA sspB*	P. Setlow (Mason and Setlow, 1986)

*The corresponding spore deficiency for the B. subtilis mutants can be found in Table 2*.

a *Cm^R^: chloramphenicol (5 μg/ml), Neo^R^: neomycin (10 μg/ml), Tet^R^: tetracycline (10 μg/ml)*.

b *German Collection of Microorganisms and Cell Cultures GmbH (Braunschweig, Germany)*.

Prior to the survival tests, the cultures were centrifuged at 4,696 × g for 5 min. in 50 ml sterile Falcon tubes. The supernatant was discarded and the pellet was resuspended in 30 ml PBS. This washing procedure was done twice.

All *Bacillus subtilis* strains used for the spore resistance experiments were congenic to their respective wild-type strain. Spores were obtained by cultivation under vigorous aeration in double-strength liquid Schaeffer sporulation medium (Schaeffer et al., 1965) under identical conditions for each strain, purified and stored as described by Nicholson and Setlow (1990). When appropriate, chloramphenicol (5 μg/ml), neomycin (10 μg/ml), or tetracycline (10 μg/ml) was added to the medium (Table 1). Spore preparations consisted of single spores with no detectable clumps, and were free (>99%) of growing cells, germinated spores and cell debris, as seen in the phase-contrast microscope (Nagler et al., 2014).

### Exposure of bacteria to abraded silicates

The cell and spore suspensions were adjusted to ~10^6^ colony forming units (CFU)/ml in PBS. For the experiments conducted in air to simulate the effect of abraded silicates inside a human habitat, the ampoules were opened by scoring and breaking of the narrow neck and the abraded silicate samples left exposed to ambient air. After 5 min exposure to ambient air, the silicate samples were mixed with the cell suspensions at a mass ratio of 1:2 (Figure 1D). The resulting slurries were vortexed and placed on a shaker at 120 rpm. Control samples were prepared in the same manner by mixing the inactivated silicate samples with the cell suspensions at a 1:2 mass ratio followed by vortexing and shaking. Experiments that were conducted under anoxic conditions to simulate the *in situ* conditions on the Martian surface were executed in the same way as the samples exposed to air except that the cell suspension were flushed with N_2_ (>99.9% purity, AGA, Denmark) prior to use to remove dissolved oxygen and that the ampoules and silicate samples were handled in a N_2_–flushed glove box. A minimum of three samples tumbled in separate ampoules were prepared for each treatment, which forms the basis for the calculated standard error of mean (SEM). All samples were exposed to air when the first subsamples were taken for plating after 15 min.

### Survival assay

The initial CFU count for each cell suspension in PBS was determined by plating in triplicates. The number of CFU was determined after 0.25, 1, 2.5, 5, and 24 h for the cell suspensions with the abraded silicates, the corresponding oxic and anoxic PBS controls as well as for the control samples with inactivated silicates. To determine the surviving fraction of cells after a given exposure time, the samples were vortexed and serial dilutions of the subsamples were plated. The survival of *D. radiodurans* and *B. subtilis* 168 was evaluated by plating on NB-plates while LB-plates were used to evaluate the survival of *P. putida* and the other *B. subtilis* strains. The plates were incubated at 30°C, and the number of colonies was determined after at least 1 day of incubation for *P. putida* and *B. subtilis* and after 2 days for *D. radiodurans* (Figure 1E). The number of colonies did not change considerably after extended incubation time. The surviving fraction was calculated as the mean number of CFU relative to the starting concentration. 100 μl of the cell suspension was streaked onto each plate giving a level of detection (LOD) of 10 CFU ml^−1^ for undiluted samples. Samples with a surviving fraction below the LOD was plotted as having a CFU count equal to the LOD. For statistical comparison, we performed unpaired *t*-tests on the log-transformed CFU counts assuming unequal variances. We had a minimum of three biological replicates for each treatment and used a significance threshold of *p* < 0.05.

### pH measurements

Inactivated quartz and basalt samples as well as abraded samples that had not been exposed to water were mixed with PBS at a 1:2 mass ratio in triplicates. The pH was measured using a glass pH electrode (Mettler Toledo Inlab® Expert Pro-ISM) with a Mettler Toledo Seven Compact pH meter after 24 h.

## Results

**Figure 2:**
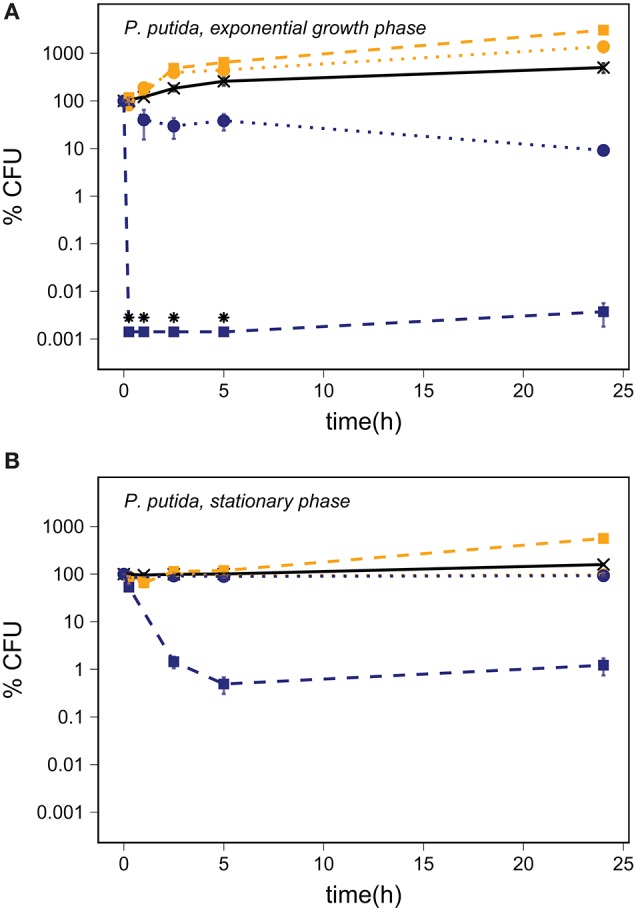
The percentage of CFU of *P. putida* harvested in the exponential growth phase **(A)** and in the stationary phase **(B)** in a PBS control (—X—) and after addition of abraded quartz (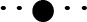) and basalt (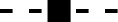) relative to the starting concentration of ~10^6^ CFU/ml. The survival of *P. putida* is shown for experiments with inactivated silicates (orange) and abraded silicates kept anoxic (blue). The error bars show the SEM, and the asterisks indicate that the number of CFU was below the LOD.

The number of CFU of *P. putida* harvested during exponential growth increased in the PBS controls as well as in the controls with inactivated silicates (Figure 2A). The abraded silicates caused a significant decrease in the number of CFU as compared to the inactivated controls with an about 99% decrease with quartz and to a concentration below the LOD with basalt (*p* < 0.001, *n* ≥ 3). Stationary phase cultures of *P. putida* cells were not affected by abraded quartz while abraded basalt killed approximately 99% of the cells within 24 h (Figure 2B), and thus caused a significant decrease in the viability as compared to the inactivated basalt (*p* = 0.008, *n* ≥ 3).

**Figure 3:**
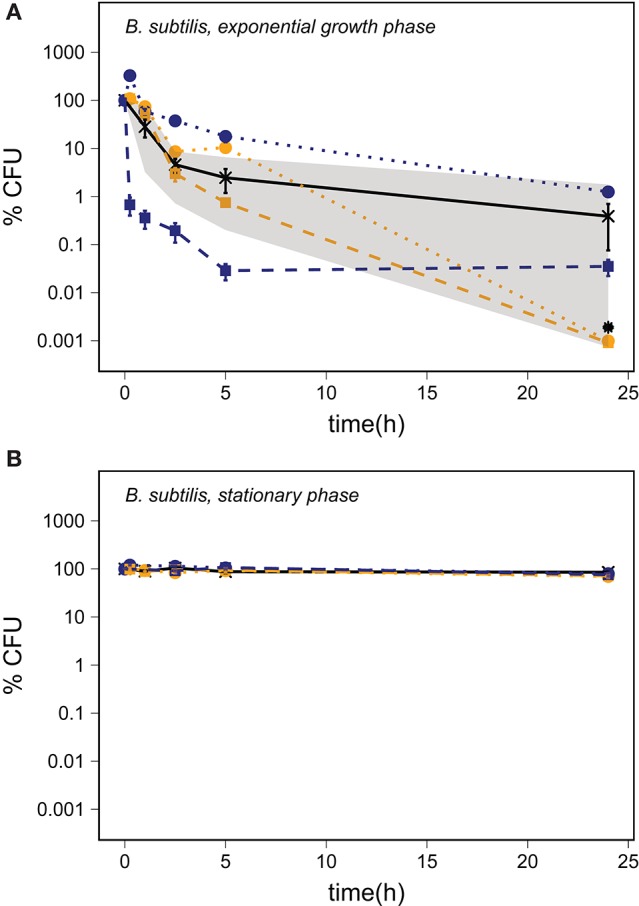
The percentage of CFU of *B. subtilis* harvested in the exponential growth phase **(A)** and in the stationary phase **(B)** in a PBS control (—X—) and after addition of abraded quartz (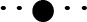) and basalt (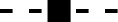) relative to the starting concentration of ~10^6^ CFU/ml. The minimum and maximum number of CFU in the PBS control replicates are marked by the gray area. The survival of *B. subtilis* is shown for experiments with inactivated silicates (orange) and abraded silicates kept anoxic (blue). The error bars show the SEM, and the asterisks indicate that the number of CFU was below the LOD.

Microscopic examination showed that the *B. subtilis* cultures harvested in the exponential growth phase were dominated by vegetative cells with no endospores observed. *B. subtilis* harvested in the stationary phase was a mixture of free endospores, endospores encapsulated by mother cells and vegetative cells without endospores. A *Bacillus subtilis* culture harvested in the stationary phase and heated to 80°C for 10 min showed a decrease to 64 ± 6% of the CFU counts before heating (average ± SEM, *n* = 3), which is in good agreement with the observed proportion of endospores. Cells of *B. subtilis* harvested in the exponential growth phase showed a substantial decrease in CFU even in the PBS control (Figure 3A). There was a faster decrease in the number of CFU in samples supplemented with abraded basalt, but the response did not differ significantly from the PBS control after 24 h of exposure (*p* = 0.70, *n* ≥ 3). The final concentration of CFU in experiments conducted with stationary phase cultures was 65-85% of the starting concentration for all treatments including the PBS control (Figure 3B).

Most of the *B. subtilis* wild-type and mutant strains showed a change in the number of CFU of less than 50% of the starting concentration following all treatments (Table 2). The only exception was PS356, which showed a decrease in the number of CFU for all treatments. This indicates that while small acid-soluble spore proteins (SASP) do not seem to be important for the resistance toward abraded basalt, they are generally important for spore survival. None of the strains had a significantly lower number of CFU after exposure to basalt as compared to both the PBS and the inactivated basalt controls (*p* ≥ 0.06, *n* = 3, for all strains).

**Table 2:** The percentage of CFU of *B. subtilis* spores relative to the starting concentration following exposure to PBS, inactivated basalt and basalt for 24 h.

**Strain**	**Gene deficiency**	**Spore deficiency**	**% CFU after 24 h**		
			**PBS**	**Inactivated basalt**	**Basalt**
PE594 (wt)	none	none	82 ± 7	76 ± 3	73 ± 5
PE620	*cotX, cotYZ*	Crust	113 ± 8	64 ± 4	64 ± 2
PE618	*cotE*	Outer coat	75 ± 7	92 ± 5	97 ± 5
PE277	*safA*	Inner coat	55 ± 3	75 ± 2	59 ± 7
PE1720	*cotE, safA*	Inner and outer spore coat	82 ± 2	113 ± 4	63 ± 5
PS832 (wt)	none	none	111 ± 5	73 ± 2	87 ± 17
PS356	*sspA sspB*	Major α- and β-type SASP	36 ± 11	25 ± 4	40 ± 2

*The basalt was kept anoxic. The results are the average of three replicates ± SEM. PE594 is the wild-type of the other PE strains while PS832 is the wild-type of the PS356 strain*.

**Figure 4:**
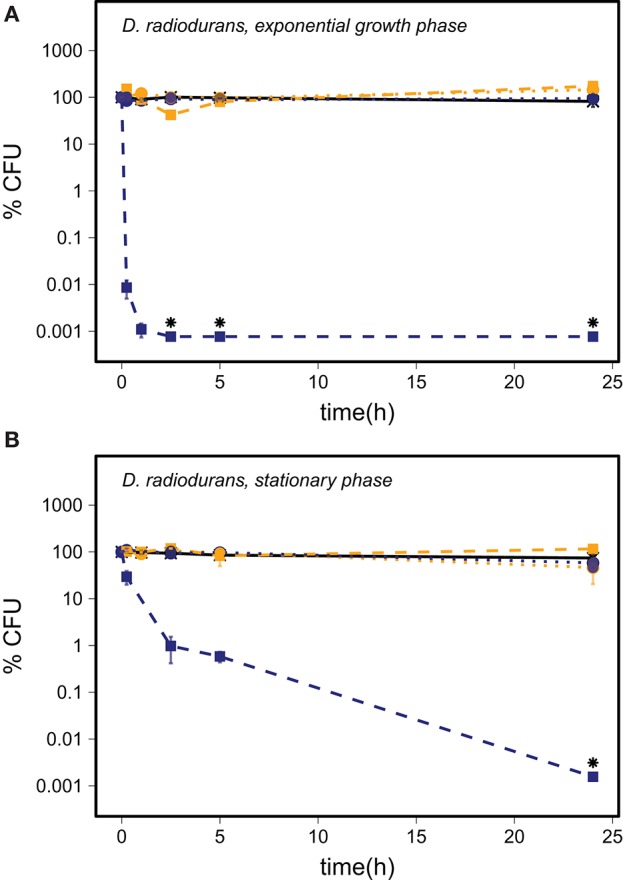
The survival of *D. radiodurans* harvested in the exponential growth phase **(A)** and in the stationary phase **(B)** in the PBS control (—X—) and after addition of abraded quartz (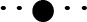) and basalt (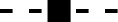) relative to the starting concentration of ~10^6^ CFU/ml. The survival of *D. radiodurans* is shown for experiments with inactivated silicates (orange) and abraded silicates kept anoxic (blue). The error bars show the SEM, and the asterisks indicate that the number of CFU was below the LOD.

The number of *D. radiodurans* CFU grown from exponential growth phase cells did not change significantly in the PBS and the quartz samples within 24 h of exposure (Figure 4A) (*p* ≥ 0.14, *n* ≥ 4). Samples exposed to abraded basalt showed the same trend as *P. putida* with an increase in the CFU number in inactivated samples and a decrease to below the LOD in activated samples (*p* < 0.001, *n* ≥ 3). Inactivated basalt did not have a considerable effect on the number of CFU of stationary phase cells while activated basalt led to a decrease in CFU number to below the LOD, although slower than for cells in the exponential growth phase (Figure 4B).

Silicate samples exposed to oxygen following the abrasion showed a lower effect on the viability of *P. putida* and *D. radiodurans* as compared to the effects of samples kept anoxic, with the exception of *D. radiodurans* harvested in the exponential growth phase and exposed to abraded quartz (Table 3). The CFU counts were in general lower than the CFU counts obtained from corresponding inactivated samples, but never decreased to below the LOD.

**Table 3:** The relative number of CFU 24 h after addition of abraded quartz or basalt (average % of initial concentration ± SEM).

**Species**	**Phase when harvested**	**PBS control**	**Inactivated quartz**	**Quartz exposed to air**	**Quartz kept anoxic**	**Inactivated basalt**	**Basalt exposed to air**	**Basalt kept anoxic**
*P. putida*	Exponential growth	503 ± 116	3, 070 ± 480	77 ± 27	9.2 ± 1.9	1, 380 ± 340	634 ± 48	below LOD
	Stationary	158 ± 7	97 ± 7	156 ± 63	93 ± 6	561 ± 35	288 ± 38	1.2 ± 0.5
*B. subtilis*	Exponential growth	0.39 ± 0.31	below LOD	below LOD	1.2 ± 0.2	below LOD	0.026 ± 0.005	0.035 ± 0.013
	Stationary	85 ± 6	70 ± 2	66 ± 5	80 ± 3	72 ± 8	69 ± 2	76 ± 7
*D. radiodurans*	Exponential growth	82 ± 19	145 ± 29	30 ± 4	94 ± 7	174 ± 15	12 ± 4	below LOD
	Stationary	74 ± 12	46 ± 26	71 ± 8	58 ± 13	116 (*n* = 1)	15 ± 12	below LOD

*A part of the data is shown as end-points in Figures 2–4*.

Addition of abraded quartz to PBS did not affect the initial pH of 7.3 ± 0.1 while addition of abraded basalt led to an increase in pH to 8.2 ± 0.1. The inactivated controls showed the same pH effect as the abraded silicates that had not been exposed to water.

## Discussion

Cells exposed to abraded silicates experience a range of potential stresses including anoxia, starvation, pH changes and ROS. The combined effect of these factors had a detrimental effect on vegetative cells as clearly seen by the experiments with abraded basalt. By including PBS controls with each experiment, we could isolate the effect of starvation and anoxia. Changes in the number of CFU due to adhesion of cells to the silicate particles or growth stimulated by supply of micronutrient should depend on the mineral composition and thus be independent of whether the samples had been inactivated or not. The inactivated silicate controls could therefore be used to account for potential effects of adhesion, supply of micronutrients as well as pH changes, which were found to be unaffected by the inactivation process. Thus, by comparing the effects of abraded silicates to the corresponding inactivated controls, we could elucidate the effects of ROS produced by the abraded silicates as well as possible direct effects of reactive surface sites produced during abrasion.

### Viability of bacteria in PBS and inactivated silicate controls

Each of the PBS controls shown in Figures 2–4 present the average of five cell cultures of which some were prepared in oxic PBS and some were prepared in anoxic PBS. We did not observe any effect of the presence of oxygen in the PBS controls and thus conclude that the oxygen regime did not directly affect the survival of the cells. Starvation, as it was induced by transferring cells from nutrient rich growth medium to PBS, affected the three bacterial species in different ways.

The increased number of CFU for exponential growth phase *P. putida* cells in PBS and in suspensions with inactivated silicate samples could partly be explained by cell division combined with a reduction in cell size. This is supported by a reduced average forward scatter and SYTO9 intensity over time (Figure S2), which reflect the overall cell size and the DNA content, respectively. Furthermore, *Pseudomonas putida* KT2440, which is identical to *P. putida* mt-2 with the exception of the presence of the TOL plasmid in mt-2 (Nakazawa, 2002), is known to accumulate large amount of polyhydroxyalkanoate (PHA) (Follonier et al., 2011). Accumulated PHA by *P. putida* mt-2 in our experiments could have provided an energy and carbon source for the additional cell divisions. The large increase in CFU number after addition of abraded basalt could indicate that growth was further enhanced by micronutrients supplied by the basalt. However, the increase in the number of CFU in the samples with inactivated quartz as compared to the PBS control remains unexplained.

The number of CFU of *B. subtilis* harvested in the exponential growth phase and transferred to PBS ranged from below the LOD to about 2% of the starting concentration after 24 h (Figure 3A). Microscopic inspection of the cells revealed that the *B. subtilis* cells became motile toward the end of the exponential growth phase and started to form endospores at the transition to the stationary phase. *Bacillus subtilis* 168 has not been shown to produce PHA (Singh et al., 2009) and, while some strains of *B. subtilis* can accumulate glycogen during sporulation with media containing *e.g*. xylose, (Kiel et al., 1994), there are no indications that *Bacillus subtilis* 168 would have accumulated storage compounds in our experiments. The sequential response to nutrient limitation with transformation into a motile stage followed by sporulation in combination with a lack of energy reserves and a sudden change to nutrient-free PBS may explain the lethal effect of starvation. The varying number of CFU after 24 h could be the result of a small but variable number of endospores in the cell suspension.

The moderate increase in the number of CFU of D. radiodurans harvested in the exponential growth phase in the inactivated silicate control samples could be due to ongoing cell division and/or separation of cell aggregates.

### The stress effect of abraded quartz

Abraded quartz did not have a considerable effect on the survival of cells harvested in the stationary phase but killed up to 91% of *P. putida* cells harvested during exponential growth (Table 3). Previous studies have shown that the addition of abraded quartz would have caused a release of about 9 μM ·OH and 31 μM H_2_O_2_ (Bak et al., 2017). This ROS production may be the cause of the observed effect as H_2_O_2_ can damage cells directly by e.g., oxidation of [4Fe-4S] clusters of dehydratases (Jang and Imlay, 2007) and mononuclear iron enzymes (Anjem and Imlay, 2012) and indirectly by production of ·OH by reactions with transition metal ions through Fenton (i.e., H_2_O_2_ + Fe^2+^ → OH^−^ + ·OH + Fe^3+^) and Fenton-like reactions (Prousek, 2007). Hydroxyl radicals react with most organic compounds at diffusion limited rates (Buxton et al., 1988) and can lead to e.g., DNA damage (Bjelland and Seeberg, 2003) and damage of cell membranes by initiating lipid peroxidation (Buege and Aust, 1978).

The bacteria used in our assays are accustomed to some level of oxidative stress as ROS constantly are produced intracellularly by adventitious reduction of molecular oxygen by reduced redox enzymes (Seaver and Imlay, 2004; Korshunov and Imlay, 2010). For *E. coli* it has been shown that these processes lead to an intracellular H_2_O_2_ concentration of about 50 nM H_2_O_2_ (Imlay, 2013) and that the production rate is equal to the influx of H_2_O_2_ when extracellular H_2_O_2_ concentrations were about 200 nM (Seaver and Imlay, 2001). An extracellular concentration of about 31 μM H_2_O_2_ following addition of abraded quartz would thus significantly increase the oxidative stress inside the cells. The increased oxidative stress would, however, not necessarily result in reduced survival as *P. putida, B. subtilis* as well as *D. radiodurans* possess a range of detoxification mechanisms.

Our test organisms can produce catalases and peroxidases and thereby effectively decompose H_2_O_2_ (Inaoka et al., 1999; Slade and Radman, 2011; Kim and Park, 2014; Svenningsen et al., 2015). Furthermore, all three species possess manganese and manganese complexes which increase the resistance to oxidative stress by scavenging ${\text{O}}_{2}^{-}$ and H_2_O_2_ and thus counteract protein damage (Inaoka et al., 1999; Horsburgh et al., 2002; Daly et al., 2010; Banh et al., 2013). Manganese can also replace iron in mononuclear enzymes (Anjem et al., 2009) which neutralizes this potential cause of Fenton-like reactions (Anjem and Imlay, 2012). Especially *D. radiodurans* is known to accumulate high concentrations of manganese, which is linked to their extreme radiation resistance (Daly et al., 2004, 2010). Also, *Deinococcus radiodurans* cells contain carotenoids, which function as antioxidants (Tian et al., 2007).

Despite this array of defense mechanisms, the cells may acquire DNA damage by oxidative stress. The vegetative cells of *P. putida, B. subtilis*, and *D. radiodurans* can counteract DNA damage by an array of DNA repair mechanisms (Slade and Radman, 2011; Lenhart et al., 2012; Mielecki et al., 2013) and especially *D. radiodurans* can withstand high degrees of DNA damage due to effective protection of the DNA repair proteins by manganese complexes (Daly et al., 2010). Even though *B. subtilis* endospores do not actively maintain the DNA while dormant, they are highly resistant to H_2_O_2_ due to the combined effect of shielding by the spore coat (Riesenman and Nicholson, 2000), low permeability of the compact inner membrane (Cortezzo and Setlow, 2005) and stabilization of DNA by α/β-type small acid soluble spore proteins (Setlow and Setlow, 1993) and dipicolinic acid (Setlow et al., 2006).

It has been shown that *P. putida* cells can survive exposure to 4 and 50 mM H_2_O_2_ in exponential phase and stationary phase, respectively (Klotz and Anderson, 1994) and the viability of *D. radiodurans* is unaffected by exposure to up to 10 mM H_2_O_2_ (Tian et al., 2007). Likewise, vegetative cells of *B. subtilis* are not affected by addition of 100 μM H_2_O_2_ (Hartford and Dowds, 1994) and endospores can resist up to 1.5 M H_2_O_2_ (Melly et al., 2002). As the expected H_2_O_2_ production from the abraded silicates is orders of magnitude lower than the H_2_O_2_ concentrations previously shown to affect the viability of *P. putida*, the observed effect on survival cannot be explained by H_2_O_2_ release. The production of ·OH from abraded quartz, however, may be responsible for the observed effects. The high reactivity of ·OH toward most organic compounds makes it virtually impossible for the cells to counteract the direct effects of ·OH.

### The stress effect of abraded basalt

Exposure to abraded basalt effectively killed both exponential growth and stationary phase cells of *P. putida* and *D. radiodurans* (Figures 2, 4). The low effect of the corresponding inactivated control samples suggests that the low viability is caused by a production of ROS from the abraded basalt samples. In a previous study, basalt samples did not lead to production of H_2_O_2_ in water, which is surprising as a fraction of the abraded material originated from abrasion of the quartz ampoules (Bak et al., 2017). It was suggested that the absence of H_2_O_2_ may be due to the presence of transition metal ions e.g., iron in the basalt, which could catalyze the breakdown of H_2_O_2_. This would entail a production of highly reactive ·OH, which may explain the greater toxicity of basalt. The significantly lesser effect of abraded basalt exposed to oxygen (Table 3) supports this hypothesis as e.g., Fe^2+^ could become oxidized by oxygen, which would counteract Fenton reactions.

Spore resistance of *B. subtilis* to abraded silicates could not be attributed to any specific spore components, as the tested mutants in general showed the same high level of resistance to abraded basalt as the wild-type strains. Based on previous studies it has been suggested that the resistance of *B. subtilis* spores to oxidizing agents is related to enzymes in the outer layers, spore coat proteins, low permeability of the compact, inner membrane and the presence of α- and β-SASPs (Setlow, 2014). While we have tested the role of the spore crust, the spore coat and α- and β-SASPs individually, we cannot exclude that the resistance is due to a combination of a range of factors for which none of the tested components are essential. If ·OH was the main agent for oxidative damage caused by abraded basalt, then lipid peroxidation could be the main cause of the effects observed with vegetative cells. In this case, we would expect the spore resistance of *B. subtilis* to be at least partly attributed to the spore layers shielding the cell membranes. This was partly tested with the mutant lacking the spore crust and the mutants lacking either the inner or the outer or both layers of the spore coat (Table 2). These mutants did, however, all maintain some parts of the outer layers of the spore, which may have been sufficient to shield the outer membrane.

### The stress effect of wind-abraded silicates on mars

Our results show that the toxicity of abraded silicates is strongly influenced by the mineralogy and secondary exposure to oxygen. The Martian soil is mainly composed of basaltic material of which the crystalline fraction is dominated by plagioclase feldspar, olivine and augite (Bish et al., 2013). This is similar to the composition of the basaltic material used in this study (Bak et al., 2017). Furthermore, under Martian *in situ* conditions the abraded basalt would only be exposed to the 10^−5^ bar oxygen present in the Martian atmosphere, which equals the oxygen concentration used for the simulated Martian atmosphere. Thus, abraded basalt that was not secondarily exposed to oxygen is the most realistic analog of the Martian soil investigated in this study. Interestingly, these samples also showed the strongest detrimental effect on survival of our test organisms.

Our exposure experiments were conducted in aqueous solution. Therefore, cells exposed to Martian soil as a result of e.g., forward contamination would not initially be exposed to the effects examined here. All known lifeforms do, however, require water for metabolic activity and ultimately to proliferate, in which case the stress effects of abraded silicates would apply. This could be circumvented by e.g., *B. subtilis* which in the form of endospores were largely unaffected by abraded silicates. Upon initial exposure to water the endospore would not be affected by the abraded silicates, and at the time of sporulation we would expect a much lower stress effect, if any, as shown by our experiments with inactivated basalt. Recent studies suggest annual formation of thin films of water on Mars at the northern water ice annulus (Kereszturi and Appere, 2014) and possibly more widespread night-time transient liquid brines caused by uptake of water vapor by deliquescent salts (perchlorates) in the soil (Martin-Torres et al., 2015). It is possible that reactive, abraded silicates accumulate in dry periods and are allowed to react periodically and locally when water is available. Abraded silicates have been found to form covalent bonds with atmospheric methane in a dry state (Jensen et al., 2014; Bak et al., 2016). Similar reactions may occur between abraded silicates and cells in a dry state, which could pose an additional challenge for life. This, however, was not investigated in the present study and awaits therefore further exploration.

The reactivity of the Martian soil is also pertinent in relation to the risk of manned missions. Freshly produced silicate dust has for long been known to cause serious health problems within the mining industry (Fubini and Hubbard, 2003), and during the Apollo missions the astronauts were complaining about respiratory irritation due to inhalation of Lunar dust brought into the spacecraft (Cain, 2010). This is partly related to physical irritation caused by inhalation of particles, but also due to production of ROS (Fubini and Hubbard, 2003; Wallace et al., 2009). The results of our experiments indicate that the reactivity of abraded silicates could be even more problematic in the dry, almost anoxic atmosphere of Mars. However, this stress effect would likely be lower inside a human habitat as secondary exposure to air led to a considerable decrease in toxicity. As shown with inactivated basalt, the effect was further reduced by exposing the abraded material to water, which could be used as simple detoxification measures.

The dramatic effects of abraded basalt on the survival of *P. putida*, and the highly radiation resistant *D. radiodurans* suggest that the Martian surface is even more hostile to terrestrial organisms than previously thought. This should lower the risk of forward contamination but may also add to the hazards encountered by future manned missions. We have not determined the cause(s) behind the toxicity of abraded silicates but put forward the hypothesis that the toxicity is the result of a production of H_2_O_2_, which through Fenton-like reactions facilitated by transition metal ions in the basalt led to the formation of highly reactive ·OH. We propose that organisms isolated from spacecraft assembly clean rooms should be examined with respect to their resistance toward exposure to abraded silicates for a better assessment of the risk of forward contamination.

## Author contributions

The study was conceived by EB, SJ, PN, and KF. EB and LJ made a pilot study for the survival assay. The experiments with *B. subtilis* mutant strains were conducted by ML and RM and the rest of the survival experiments were performed by EB. SN conducted the flow cytometry analyses. The manuscript was written by EB and KF with input from ML, SN, LJ, RM, SJ, and PN.

### Conflict of interest statement

The authors declare that the research was conducted in the absence of any commercial or financial relationships that could be construed as a potential conflict of interest.
