# Supplementary material for: Silicates Eroded under Simulated Martian Conditions Effectively Kill Bacteria—A Challenge for Life on Mars
Source: Front Microbiol. 2017 Sep 12;8:1709. doi: 10.3389/fmicb.2017.01709 (PMC5601068; doi:10.3389/fmicb.2017.01709)
Supplement: Supplementary file 1 [file DataSheet1.docx]

Supplementary Material

Silicates eroded under simulated Martian conditions effectively kill bacteria - a challenge for life on Mars

Ebbe Norskov Bak^1*^, Michael Goul Larsen^1^, Ralf Moeller^2^, Silas Boye Nissen^1^, Lasse Riis Jensen^1^, Per Nørnberg^1^, Svend J. Knak Jensen^3^, Kai Finster^1,4^

^1^Department of Bioscience, Aarhus University. Ny Munkegade 116, 8000 Aarhus C, Denmark

^2^Institute of Aerospace Medicine, Space Microbiology Research Group, German Aerospace Center (DLR e.V.), Linder Höhe. D-51147 Cologne (Köln). Germany

^3^Department of Chemistry, Aarhus University. Langelandsgade 140, 8000 Aarhus C, Denmark

^4^Stellar Astrophysics Center, Department of Physics and Astronomy, Aarhus University. Ny Munkegade 120, 8000 Aarhus C, Denmark

*** Correspondence:**

Ebbe Norskov Bak

ebbe.bak@bios.au.dk

# Supplementary Figures and Tables


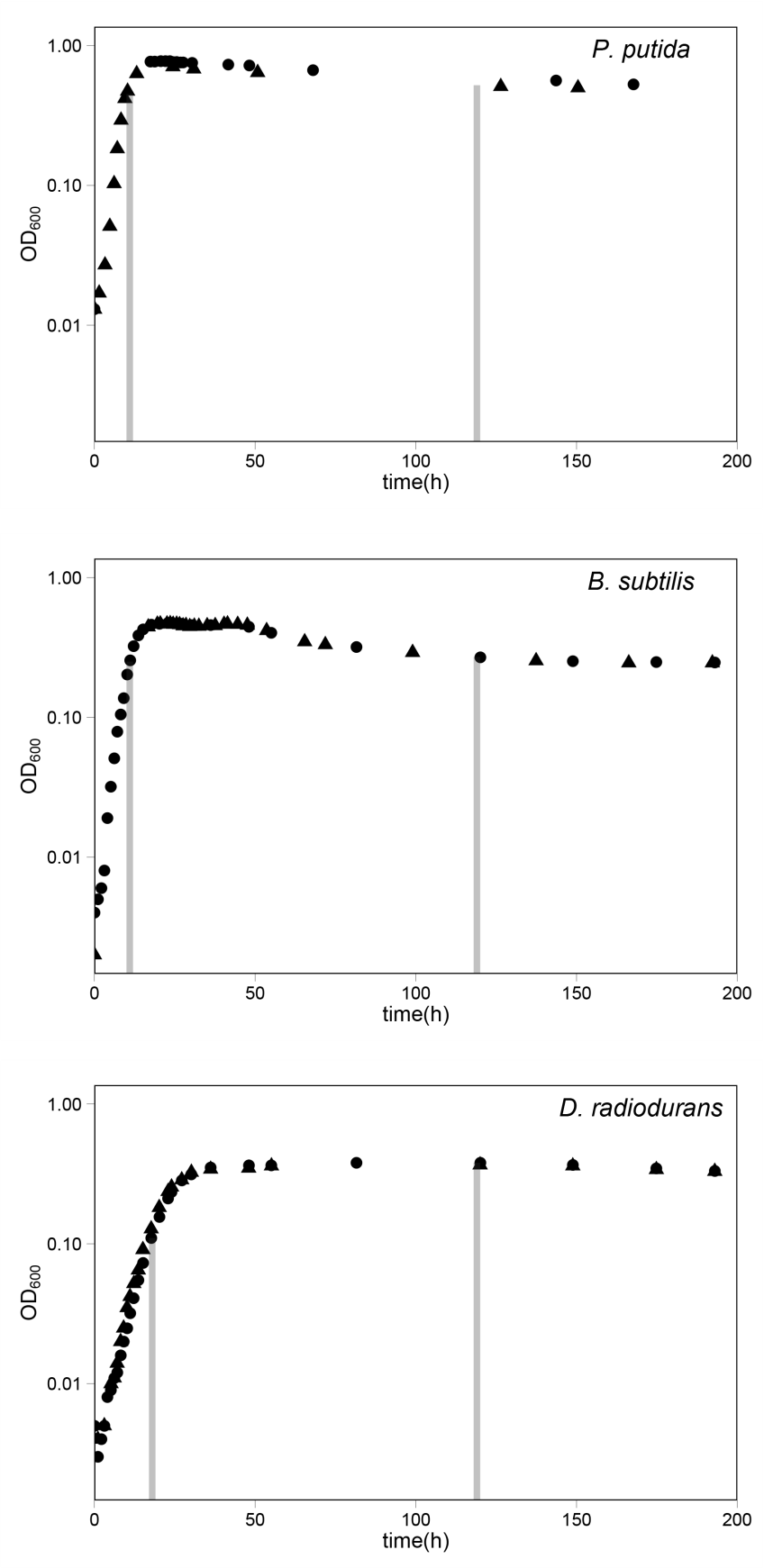
**Figure S1. Growth curves for *P. putida, B. subtilis* and *D. radiodurans*.** The cultures were incubated in 30 ml of 20% LB media and 80% PBS for *P. putida* and 30 ml of 20% NB media and 80% for *B. subtilis* and *D. radiodurans* in 100 ml Erlenmeyer flasks on a shaker running at 120 rpm at 21°C. The flasks were fused with test tubes so that we could direct the media to the test tube and measure the OD_600_ without taking out subsamples. The OD_600_ was measured on a Pharmacia Biotech Novaspec II calibrated with pure media of the same type. The triangels and the circles show the results for two biological replicates. The gray bars show the sampling times for the bacteria during growth (10-12 hours for *P. putida* and *B. subtilis* and 17-19 hours for *D. radiodurans*) and for the bacteria in late stationary phase (118-120 hours for all three species).








**Figure S2.** A: Average forward scatter and B: SYTO9 intensity of *P. putida* cells in PBS. Exponential growth phase (blue) and stationary phase (black). The error bars show the standard deviation.

# Supplementary methods

## Flow cytometry

Subsamples of 300 μl from *P. putida* cultures in PBS were transferred to 5 ml Falcon tubes and mixed with one μl of a 1:1 mixture of SYTO9 and propidium iodide (PI) from the LIVE/DEAD® BacLight™ Bacterial Viability Kit L7012. The samples were incubated in the dark for 15 min at 21-23°C and 50 μl of each sample were analyzed on an ACEA Biosciences NovoCyte™ flow cytometer. The flow cytometer was set to a flow rate of 30 μl min^-1^ and a core diameter of 11.3 µm. The forward scatter and the SYTO9 intensity (excitation 488 nm, emission 530 nm) for each cell/particle were recorded. The data were analyzed using FlowJo v. 10.0 (FlowJo LLC, Asland, OR).
